# A rare tonsillar metastasis of Merkel cell carcinoma: A case report

**DOI:** 10.1002/ccr3.7063

**Published:** 2023-03-19

**Authors:** Precious C. Okonkwo, Ania Henning, Jose Plaza, Amit Agrawal, Carlo M. Contreras

**Affiliations:** ^1^ Kent State University Kent Ohio USA; ^2^ The Ohio State Wexner Medical Center Columbus Ohio USA

**Keywords:** cancer, metastasis, skin, tonsil

## Abstract

Merkel cell carcinoma (MCC) metastasis to the tonsil is extremely rare with five published cases. We report the case of a patient with palatine tonsillar MCC metastasis, who presented without oropharyngeal symptoms, which contrast prior reported cases.

## Abstract 2

Merkel cell carcinoma (MCC) metastatic to the palatine tonsil is extremely rare with five published cases. We report an isolated, asymptomatic distant metastasis to the tonsil elucidated by preoperative imaging for a node‐positive buttock MCC, which contrast prior reported cases. This condition was treated with resection of the primary tumor, regional lymphadenectomy, and tonsillectomy followed by adjuvant immune checkpoint inhibitor therapy.

## 1 INTRODUCTION

Merkel cell carcinoma (MCC) is a rare, aggressive neuroendocrine tumor that commonly presents as cutaneous or mucosal tumor, arising from Merkel mechanoreceptor cells in the skin and in ectoderm‐derived mucosa.^1^, ^2^ MCC has been reported to have a rising incidence rate of 95% from 2000 to 2013.^3^ Despite the early detection and optimal control with surgical and adjuvant radiotherapy for Stage I–III disease, the overall survival rate is between 30% and 75%.^4^, ^5^ MCC development is associated with Merkel cell polyomavirus (MCPyV) in 80% of cases and chronic UV‐mediated damage in 20% of cases, and commonly seen among people older than 50 years.^6^, ^7^ Studies have identified the most frequently affected areas to be the head and neck region (50%), trunk (30%) and the arms and legs (10%), and approximately 4.5% are of mucosal origin.^3^, ^7^ About 65%–70% of patients with MCC present with localized disease to the skin, 25% have regional lymphadenopathy, and 5%–8% have distant metastasis with the most common sites being the liver, lung, brain, and bone.^8^, ^9^

Adults are more likely than children to have MCPyV detectable by polymerase chain reaction in elective tonsillectomy specimens obtained for non‐oncologic indications.^10^ Despite this, tumor metastatic to the oral cavity is an infrequent occurrence, with metastasis to the palatine tonsil being an extremely rare event.^2^, ^11^ Considering all primary tumor types, only about 100 cases of metastases to the palatine tonsil have been documented in the literature, specifically with MCC metastatic to the palatine tonsil comprising of only five of the cases.^1^, ^2^ Among the reported cases of metastatic MCC to the palatine tonsil, patients commonly presented with pharyngitis, dysphagia, and other oropharyngeal symptoms, years after wide excision of the primary cutaneous tumor.^1^, ^2^, ^5^

We report a case of a 57‐year‐old female patient who presented with primary MCC of the right buttock, with an incidental, asymptomatic metastasis to the left palatine tonsil.

## 2 CASE PRESENTATION

An otherwise healthy 57‐year‐old female patient presented with a 3‐year history of an enlarging right buttock soft tissue mass. The mass was not associated with pain; however, there was a history of minor trauma to the area. Physical examination revealed a right buttock cutaneous mass measuring 12 × 8 cm and a firm, non‐tender right inguinal mass. Pre‐referral computed tomography (CT) of the pelvis with IV contrast demonstrated a lobulated 8 cm right buttock mass and multiple enlarged right inguinal lymph nodes. Core needle biopsy of the buttock and fine needle aspirate of the inguinal mass demonstrated neuroendocrine carcinoma.

**FIGURE 1:**
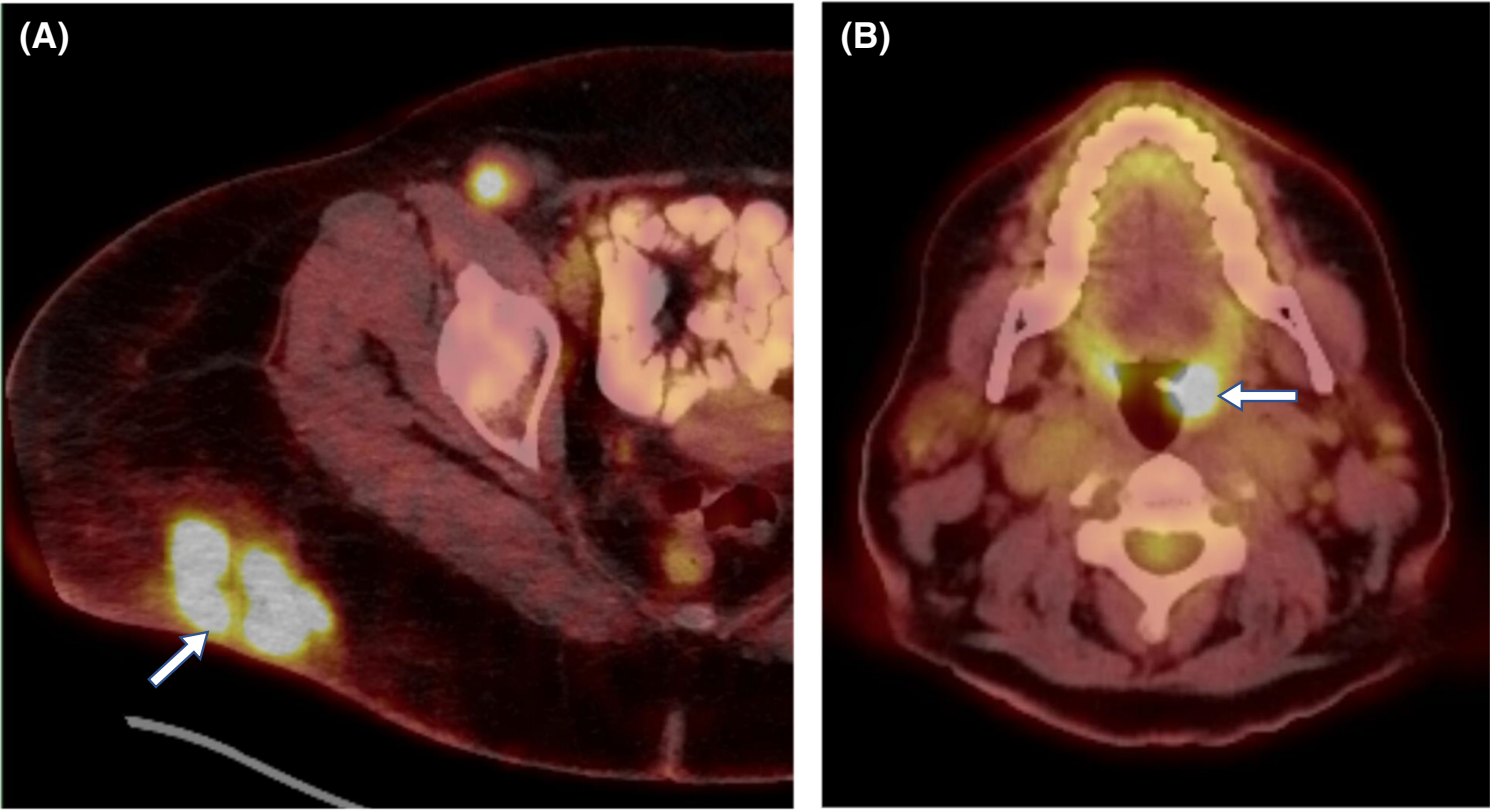
Cross‐sectional PET‐CT images showing fluorodeoxyglucose accumulation in the right buttock (arrow in A), indicating the tumor, and asymmetric accumulation in the left tonsil (arrow in B), concerning for neoplasm or inflammatory process.

Immunohistochemical staining of the right buttock diagnostic biopsy specimen was performed, which was positive for CK20, chromogranin, EMA and Merkel cell polyoma virus and negative for thyroid transcription factor‐1 (TTF‐1) and CK7. These findings were consistent with the diagnosis of MCC. Further examination with MCPyV oncoprotein antibody test was obtained, revealing an antibody titer of 115,000 standard units. At our institution, fused fluorodeoxyglucose‐18 positron emission tomography (PET)‐CT scan noted asymmetric uptake involving the left tonsil region, concerning for neoplasm versus an inflammatory process (Figure 1). She denied associated oropharyngeal symptoms and a firm left tonsil was noted on oral examination; there was no palpable cervical or supraclavicular lymphadenopathy.

**FIGURE 2:**
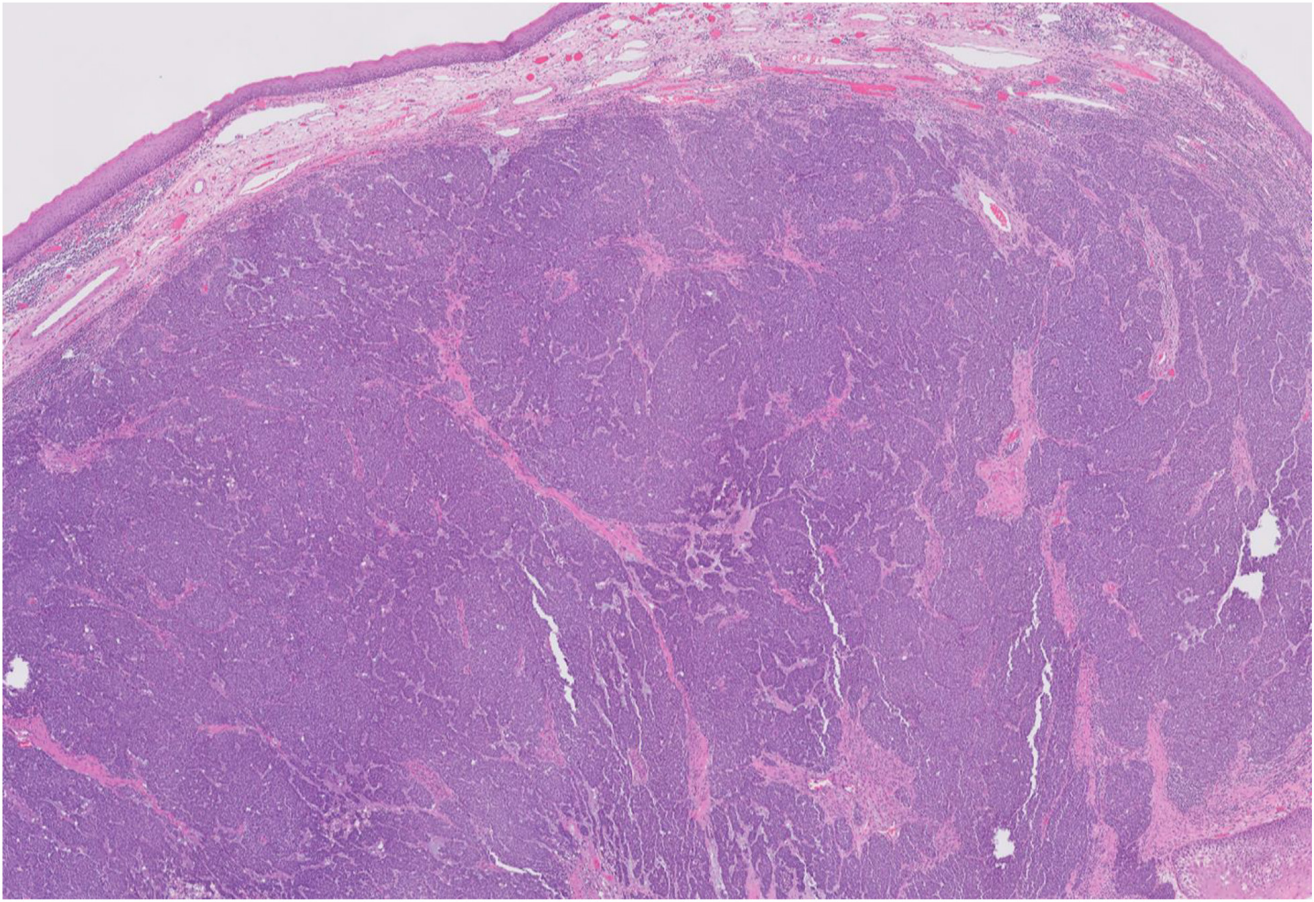
Hematoxylin and eosin stain (10×) of tonsil showing a nodular proliferation of small blue cells with scant cytoplasm, indistinct nucleoli, and finely dispersed chromatin forming diffuse sheets and completely replacing normal tonsillar parenchyma.

**FIGURE 3:**
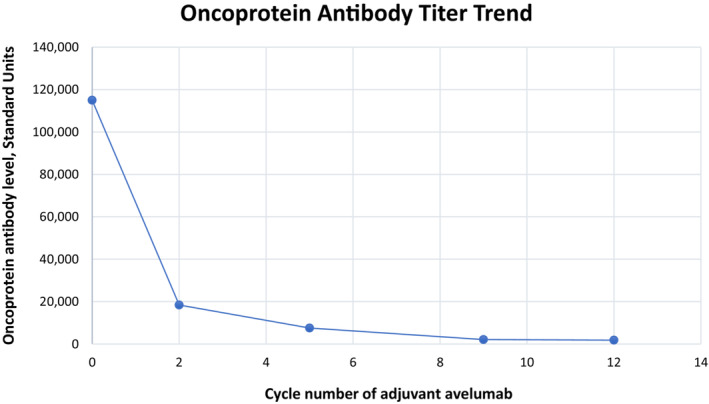
Graphical representation of the serum MCPyV oncoprotein antibody titer trend.

Per the evidence‐based^4^ consensus recommendation of our multidisciplinary cutaneous tumor board, the patient underwent left tonsillectomy, right buttock wide local excision, and lymphadenectomy of her right inguinal, iliac, and obturator basins with sartorius muscle transposition flap coverage of the femoral vessels. Consideration was given to preoperative tonsillar biopsy, but this was not pursued due to the risk of bleeding and sampling error. Postoperatively, she was discharged in good condition after an uncomplicated 4‐day inpatient stay. Evaluation of her surgical specimens demonstrated a 6.5 cm MCC of the right buttock with widely negative microscopic margins, mitotic index of 22 mitoses per mm^2^ and lympho‐vascular invasion; 1/16 lymph nodes involved; and metastatic MCC of the left tonsil (Figure 2). One month later, she underwent a planned second‐stage rotational flap closure of the gluteal resection defect. Her final pathologic staging was Stage IV (pT4N1bM1c) and she initiated a 1‐year course of adjuvant therapy with avelumab 800 mg every 2 weeks. On follow‐up visits, a decreasing trend in her MCPyV oncoprotein antibody titer was noted, as shown in Figure 3.

The patient is currently 11 months from the initial diagnosis of MCC of the right buttock, 10 months from her pathological diagnosis of metastatic MCC of the tonsil, and is without any evidence of disease progression based on physical examination and contrast‐enhanced surveillance CT imaging. She continues her course of adjuvant avelumab therapy and is closely monitored for disease progression with regular follow‐ups.

## 3 DISCUSSION

Tonsillar neoplasm is a rare occurrence and metastasis is extremely rare, with 0.8% accounting for metastasis with extra‐tonsillar primary.^12^ Metastatic tonsillar tumors are uncommon due to the fact that the tonsil lacks afferent lymphatic vessels and histologically consists of mainly reticuloendothelial cells with strong ability to clear tumor.^13^ Therefore, in this case, malignant dissemination to the tonsil is suggested to be by hematogenous route.^5^ An alternative route would be retrograde via cervical lymphatics to the tonsil.^5^, ^14^

Colorectal carcinoma, breast cancer, lung cancer, gastric carcinoma, melanoma, and seminoma are the primary tumors that account for the majority of metastases to the palatine tonsil.^12^ Metastatic involvement to the tonsil is typically metachronous and generally presents with odynophagia, pharyngitis, dysphagia, and globus.^13^ The five prior reported cases of metastatic MCC to the palatine tonsil are metachronous (7 months to 7 years after presentation) and were all symptomatic.^1^ In one study, the authors used microarray‐based comparative genomic hybridization to demonstrate that what appeared to be a metachronous metastasis to the tonsil was actually a second primary MCC.^15^ In contrast, we report a patient presenting with a synchronous, asymptomatic palatine tonsillar metastasis that was incidentally discovered on staging PET‐CT.

MCC risk factors include chronic UV‐mediated damage to sun‐exposed areas of the skin and chronic immunosuppression due to HIV, lymphoma, or organ transplantation.^8^ Microscopically, MCC has similar histopathological characteristics as small‐cell carcinoma of the lungs—small, blue, round cells with prominent nuclei and scant cytoplasm, and must be excluded with a negative TTF‐1 and CK7, which was noted in our case.^6^, ^8^ Other immunohistochemical staining that are helpful in differential diagnosis include: positive CD99 for primitive neuroectodermal tumor, leukocyte common antigen positive for small‐cell lymphomas and S100, Melan‐A and HMB‐45 positive for malignant melanoma.^8^ As a neuroendocrine tumor, neuroendocrine markers such as chromogranin A, synaptophysin, CD56, neuron‐specific enolase usually confirm the diagnosis.^1^, ^16^ MCPyV involvement in MCC is demonstrable via tumor staining and serology (80% and 60%, respectively).^8^ Though MCC in patients with elevated MCPyV oncoprotein antibody titers have a favorable survival compared to seronegative patients, and when detectable, the oncoprotein antibody titer can serve as a tumor marker indicative of overall MCC burden.^17^ In this case, the serum MCPyV oncoprotein antibody was elevated at diagnosis and appropriately continues to decrease throughout the treatment course. The efficacy of immune checkpoint inhibition in patients with advanced MCC is thought to relate to the high tumor mutation burden, as the MCPyV status is not predictive of response to immunotherapy.^17^, ^18^

## 4 CONCLUSION

Distant metastasis of MCC to the palatine tonsil is extremely rare. The reported cases involve patients with symptomatic, metachronous presentation. This current report is the first to present an asymptomatic, synchronous case of metastatic MCC to the tonsil. Due to the natural history of this tumor, aggressive multimodal treatment is crucial.

## AUTHOR CONTRIBUTIONS

**Precious C Okonkwo:** Conceptualization; writing – original draft. **Ania Henning:** Writing – review and editing. **Jose A Plaza:** Writing – review and editing. **Amit Agrawal:** Writing – review and editing. **Carlo M Contreras:** Supervision; writing – review and editing.

## FUNDING INFORMATION

No funding source to be declared.

## CONFLICT OF INTEREST STATEMENT

Authors have no conflict of interest to declare.

## CONSENT

Written informed consent was obtained from the patient to publish this report in accordance with the journal's patient consent policy.

## Data Availability

The data presented in this study are available in this article.

## References

[1] DeGiovanni JC , Kuo CC , Tokarz EL , Shah AT , O'Leary M . Not your typical tonsil: metastatic Merkel cell carcinoma or primary disease? Cureus. 2021;13(4):e14604. 34040905 10.7759/cureus.14604 PMC8139134

[2] Vasileiadis I , Sofopoulos M , Arnogiannaki N , Georgopoulos S . A Merkel‐cell carcinoma metastatic to the tonsil: a case report and review of the literature. J Oral Maxillofac Surg. 2013;71(10):1812. 10.1016/j.joms.2013.06.196 23911145

[3] Paulson KG , Park SY , Vandeven NA , et al. Merkel cell carcinoma: current US incidence and projected increases based on changing demographics. J Am Acad Dermatol. 2018;78(3):457‐463. 29102486 10.1016/j.jaad.2017.10.028 PMC5815902

[4] Bichakjian CK , Olencki T , Alam M , et al. Merkel cell carcinoma, version 1.2014. J Natl Compr Cancer Netw. 2014;12(3):410‐424. 10.6004/jnccn.2014.0041 PMC9441108 24616545

[5] Tesei F , Farneti G , Cavicchi O , Antonelli P , Zanetti G , Leone O . A case of Merkel‐cell carcinoma metastatic to the tonsil. J Laryngol Otol. 1992;106(12):1100‐1102. 1487674 10.1017/s0022215100121899

[6] Uchi H . Merkel cell carcinoma: an update and immunotherapy. Front Oncol. 2018;8:48. 29560342 10.3389/fonc.2018.00048 PMC5845720

[7] Kouzmina M , Koljonen V , Leikola J , Böhling T , Lantto E . Frequency and locations of systemic metastases in Merkel cell carcinoma by imaging. Acta Radiologica Open. 2017;6(3):2058460117700449. 28540062 10.1177/2058460117700449 PMC5433554

[8] Açıkalın A , Paydaş S , Güleç ÜK , Uğuz A , Gümürdülü D . A unique case of Merkel cell carcinoma with ovarian metastasis. Balkan Med J. 2014;31(4):356‐359. 25667793 10.5152/balkanmedj.2014.14117 PMC4318410

[9] Gillenwater AM , Hessel AC , Morrison WH , et al. Merkel cell carcinoma of the head and neck: effect of surgical excision and radiation on recurrence and survival. Arch Otolaryngol Head Neck Surg. 2001;127(2):149‐154. 11177031 10.1001/archotol.127.2.149

[10] Kantola K , Sadeghi M , Lahtinen A , et al. Merkel cell polyomavirus DNA in tumor‐free tonsillar tissues and upper respiratory tract samples: implications for respiratory transmission and latency. J Clin Virol. 2009;45(4):292‐295. 19464943 10.1016/j.jcv.2009.04.008 PMC7172143

[11] Gangwani P , Sadda A , Danakas A , Kolokythas A . Metastatic disease to the condyle: a case report and review of literature. Oral Maxillofac Surg Cases. 2022;8(1):100244.

[12] Zaubitzer L , Rotter N , Aderhold C , et al. Metastasis of pulmonary adenocarcinoma to the palatine tonsil. Mol Clin Oncol. 2019;10:231‐234. 30680199 10.3892/mco.2018.1776 PMC6327221

[13] Sera T , Kashiwagi S , Takashima T , et al. Multiple metastatic malignant phyllodes tumor of the breast with tonsillar metastasis: a case report. BMC Res Notes. 2017;10:55. 28103951 10.1186/s13104-017-2375-5 PMC5248444

[14] Wang H , Chen P . Palatine tonsillar metastasis of rectal adenocarcinoma: a case report and literature review. World J Surg Oncol. 2013;11(1):114. 23705669 10.1186/1477-7819-11-114 PMC3669029

[15] Nagy J , Fehér LZ , Sonkodi I , Lesznyák J , Iványi B , Puskás LG . A second field metachronous Merkel cell carcinoma of the lip and the palatine tonsil confirmed by microarray‐based comparative genomic hybridisation. Virchows Arch. 2005;446(3):278‐286. 15731927 10.1007/s00428-004-1176-0

[16] Visscher D , Cooper PH , Zarbo RJ , Crissman JD . Cutaneous neuroendocrine (Merkel cell) carcinoma: an immunophenotypic, clinicopathologic, and flow cytometric study. Mod Pathol. 1989;2(4):331‐338. 2668940

[17] Chan TA , Yarchoan M , Jaffee E , et al. Development of tumor mutation burden as an immunotherapy biomarker: utility for the oncology clinic. Ann Oncol. 2019;30(1):44‐56. 30395155 10.1093/annonc/mdy495 PMC6336005

[18] Spassova I , Ugurel S , Kubat L , et al. Clinical and molecular characteristics associated with response to therapeutic PD‐1/PD‐L1 inhibition in advanced Merkel cell carcinoma. J Immunother Cancer. 2022;10:e003198. 35074902 10.1136/jitc-2021-003198 PMC8788332

